# Address

**Published:** 1897-06

**Authors:** T. G. Rix


**THE DENTAL REGISTER.**

VOL. LI.] JUNE, 1897. [No. 6.

### COMMUNICATIONS.

### Address.

BY DR. T. G. RIX.

President of the South Western Dental Society of Michigan, April 15th, 1897.

Gentlemen:—I will not annoy you with "a long, formally written" address, but will merely occupy a few moments time in giving the young members of the profession a few points in dental ethics, and a little fatherly advice from the standpoint of an *old timer*, perhaps you will say "an old fogie," or a "has been." Let that go as it may, I've got this chance to have *my say*, and I'll say it. Perhaps you will kick, but so be it.

My young friends, you who are just starting out in professional life, fondly hoping to make for yourselves fame and fortune, do not worship the *Almighty Dollar* to the neglect of your professional manhood. Rather sacrifice a little of the *pelf* than a large amount of professional honor. Never wish for a dollar without giving an equivalent. Seek first to benefit your fellow man. If you do this conscientiously all else will be given unto you.

In all undertakings in life, in whatever calling, three things are absolutely necessary to success, namely: Adaptability, qualification and liking. You surely must fail if you can not adapt yourself to your calling by a natural or acquired *tact* to do that which you are called upon to do; and a qualification by education and practice which shall give confidence to the brain and skill to the hands. Love is the fulfilling of the *law*, and if you have not love for your chosen profession, you are lacking in one of the essentials to success. Not that love that shall be vain and puffed up over the seeming respectibility of the calling, but as a means to an end. Loving it, because through it you are enabled to benefit humanity, and relieve human suffering. I have said many times during my professional life (and I assure you I meant it), that if 'twere only for the dollars and cents I would not practice dentistry a day; for who will dispute the fact that it is one of the most fatiguing, nerve enfeebling, disagreeable professions in the world.

Of course there are a few pleasant, as well as the many disagreeable things. I will not stop to particularize, but in all candor I will appeal to you who have borne the burden and heat of the day in dentistry. If knowing what you do, after the many years in the harness, would you desire to spend another lifetime in the service, for the mere dollars and cents that go with it?

There may be those who care not for humanity or human obligations. But every honest, conscientious dentist, will first consider his patients interests and welfare before he does his own. Be truthful and honest with your patients; never desiring their money unless you can give an equivalent; you should refuse to serve them if you know you cannot benefit them, otherwise it would simply be *robbery*. Don't think for a moment that when I know I have greatly benefited a person and I know they know it, that I would not charge them a good fee. No, in such a case you can justly and conscientiously charge a fee commensurate to the services rendered, and their ability to pay. Under such conditions you will have no "kick" and the bills will be paid with pleasure and many thanks.

You will find those who will not appreciate your services at any price. To all such give a wide berth, never caring to serve them except may be to relieve them temporarily; let them alone as much as possible, they will do you no good or themselves either. "It is like casting pearls before swine, that may turn and rend you."

Never let a person come *shopping* on you, nor get you to set a price on your services, lest you might be induced to slight them on account of the low fees quoted. Rather give them to understand that unless they preferred you to any one else and had perfect confidence in your ability and integrity, you would not care to serve them at any price. Make these presentations with kindness and affibility, and you will be surprised at the effect it will have on them, and what a change will generally be produced in their demeanor toward you. They will soon be anxious to get appointments with you and come to be your solid patrons. There are a few truisms as applicable to dentistry as to any other calling, perhaps more so. One is, "That which is worth doing at all, is worth well doing."

You can not afford to do poor work at any price, neither can you afford to do good work for nothing, your professional reputation and standing depends on your skill; therefore, in every instance *do your best*, the best will be poor enough. You will meet with many failures, but should you discover them in others you should cover their faults, foibles and errors with the mantle of charity, as much as you can conscientiously, never commenting on them or exposing them to their patients. It would be unneighborly, unprofessional and unkind. It would also be impolitic; your chickens may come home to roost too soon, for your own comfort.

I have found as an old german friend used to say, in his quaint, wise-looking way, "The longer I lives, the more by——I finds oud." I have found out that if we live long enough, and practice in the same community we will find failures of our own "oud," and many of them at that. If a dentist should see fit to hang out his shingle in the locality where you have practiced for years, don't greet him with a look or manner of a pre-emptionist of locality and fee simple owner of the earth sort of way, but instead, give him a fraternal hand, be kind and accommodating,don't try to make him think you're the only "Pebble on the Beach," he would'nt believe you if you did, for "There are others," don't give him the marble heart and icy mitt, but try to draw him to you by kindness, and instead of his being a thorn in the flesh he may be a help-mate and friend in times of need. Cast your bread upon the waters, etc.

My advice to every young dentist is (in order to keep well abreast of the times) to visit, commune and associate with your professional brethren on every possible opportunity, make frequent visits with the leading lights, and attend all the society meetings, where you will hear discussed all subjects pertaining to the art.

In my nearly forty years in dentistry, association has done more to broaden and round out whatever knowledge and skill in the art I otherwise might have possessed. Therefore, never fail, whenever the opportunity presents itself, to call on your brother dentist. Approach him in the right spirit and you are sure to draw him out and get many new and valuable ideas. Should you happen to approach him in an arrogant, "Know it all" manner, you are apt to get left, he will probably shut up like a clam and you will undoubtedly come away feeling that you have been badly snubbed and insulted, when in fact you were the one more to blame.

Modesty is more becoming (in one seeking knowledge) than assumption; therefore, approach in humility as one seeking to learn. Lead him to feel that you consider him if not wiser, fully as wise as yourself, and you will be surprised to see how quickly he will unbend, though he be *savant* or *fool*. In either case you are sure to learn something which will be of use to you sooner or later. I never yet ran across a dentist so ignorant as to preclude the possibility of my learning something from him, and getting some good out of him. Therefore, let us not despise the day of small things, for we may entertain an angel (or wise dentist) unawares.

Before I sit down, it will perhaps be fitting in me to call your attention to the death of one of our charter members, Dr. Ray, of St. Joseph, his death was a sad blow to me, having known him quite well for 25 years. He was a bright man in his profession, yet modest and unassuming. He was a warm and true friend, a fond husband and indulgent father, I would recommend that a fitting testimonial of his worth be presented at this meeting.

